# Perioperative dexamethasone in high-grade gliomas: the short-term benefits and long-term harms

**DOI:** 10.3389/fonc.2023.1335730

**Published:** 2023-12-14

**Authors:** Akshitkumar M. Mistry

**Affiliations:** Department of Neurological Surgery, University of Louisville, Louisville, KY, United States

**Keywords:** dexamethasone, glioblastoma, glioma, survival, immunosuppression

## Abstract

Dexamethasone has been commonly given to patients with a presumed new GBM in relatively large doses (6-16 mg daily for 1-2 weeks) since the 1960s without any rigorous evidence. This treatment with dexamethasone before the diagnosis and adjuvant therapy makes GBM patients unique compared to other newly diagnosed cancer patients. While dexamethasone may be beneficial, recent studies suggest that this potent immunosuppressant with pleiotropic effects is harmful in the long term. This perspective article summarizes the disadvantages of perioperative dexamethasone from multiple facets. It concludes that these growing data mandate rigorously testing the benefits of using perioperative dexamethasone.

## Introduction

Glioblastoma (GBM) is the most common, malignant, and therapy-resistant brain tumor (1). The median overall survival of GBM patients is stagnated around 15 months (2–4). Although there have been small studies showing benefit like lomustine-temozolomide combination for GBMs with methylated *MGMT* promoter (5) (a larger trial is underway, NCT05095376) and immunotherapy for GBMs with high mutational burden (6), besides surgical resection, only three GBM therapies have rigorous practice-changing data: radiation [1980 (7)], temozolomide chemotherapy [2005 (3)], and alternating electric fields [2017 (8)]. Countless other therapies with groundbreaking success in other cancers have failed to improve the survival of GBM patients. The recent failure of the revolutionary immune checkpoint inhibitors (2, 9, 10) now poses an urgent question: Is therapy failure iatrogenic? To answer, we must reexamine the common GBM treatment, which begins with a diagnostic surgery, then after about 3 weeks, adjuvant treatment with the above three therapies. Intriguingly, before and after the surgery (i.e., perioperatively), dexamethasone is commonly given to patients with a presumed new GBM in relatively large doses (6-16 mg daily for 1-2 weeks) (11, 12). This treatment with dexamethasone *before* the diagnosis and adjuvant therapy makes GBM patients *unique* compared to other newly diagnosed cancer patients. Dexamethasone is a potent immunosuppressive steroid with pleiotropic effects (13).

## Benefits of dexamethasone

Perioperative dexamethasone decreases tumor- and surgery-associated symptoms. Symptoms in GBM patients arise from the brain directly injured or replaced by the tumor and the surrounding ‘normal’ brain that is compressed by the tumor and becomes edematous in response, contributing to the increased intracranial pressure. Dexamethasone mitigates symptoms by controlling this edema. Mechanistically, it is a long-acting synthetic corticosteroid that decreases microvascular permeability by reducing vascular response to and expression of tumor-derived permeability factors like vascular endothelial growth factor (VEGF) (14–16). Additionally, preoperative dexamethasone treatment is believed to decrease brain swelling during GBM surgery (17, 18). Postoperatively, dexamethasone is commonly continued as a high-dose, 2-week taper because of its anti-inflammatory and psychiatric effects. It lowers the normal inflammation caused by surgery and mitigates symptoms such as pain and nausea. Therefore, despite having a major operation, patients feel vigorous, are more active, leave the hospital earlier (19), and show better performance status at the start of adjuvant therapy (20). Because dexamethasone is very potent, these effects can be readily observed. Thus, since the 1960s (21), it has been used with no rigorous evidence (22–24).

## Long-term harms of dexamethasone

**Figure 1:**
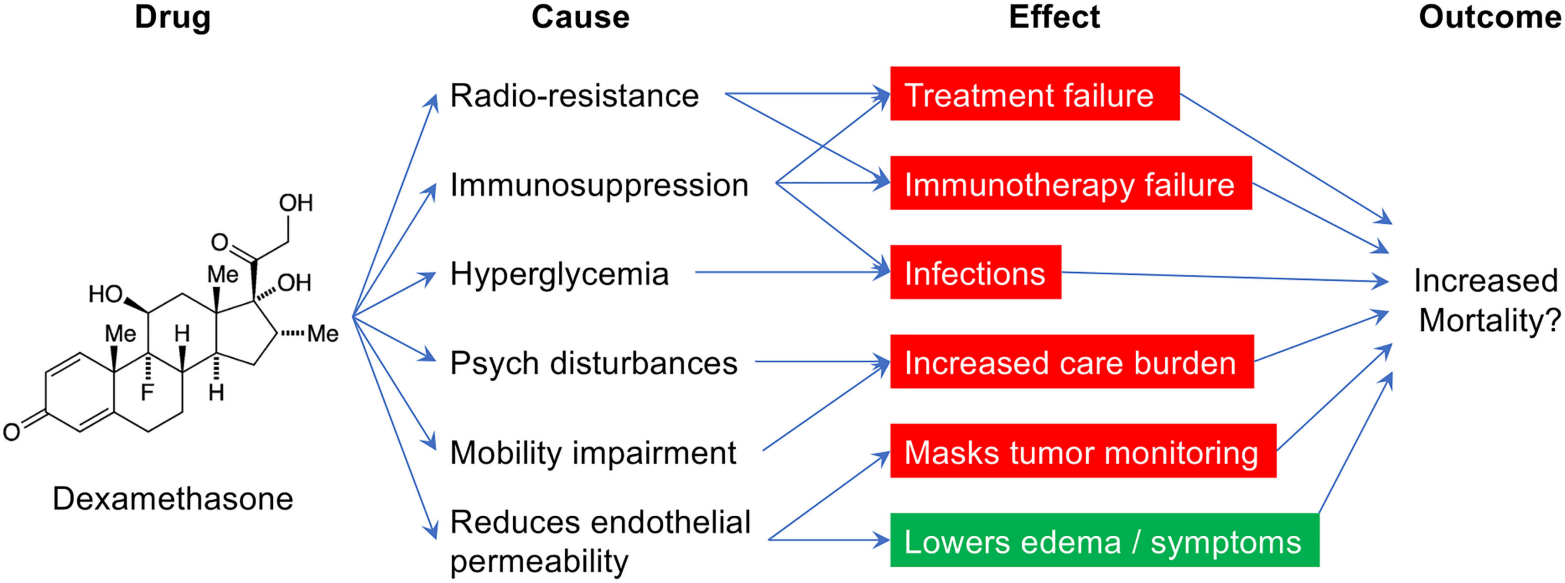
The various causes and potential effects of perioperative dexamethasone in glioblastoma patients.

Perioperative dexamethasone *may* be beneficial but concerns for its long-term harm are growing (**Figure 1**).

Dexamethasone’s psychiatric and cognitive effects are deleterious (25). It increases the risk of postoperative insomnia, irritability, mania, and delirium, which can lead to early mortality (13, 26–28). A large, multicenter, phase 3 randomized controlled trial (RCT) showed significantly greater psychiatric disorders within 30 days in subdural hematoma neurosurgery patients randomized to a 2-week course of dexamethasone (29). Long-term use of dexamethasone leads to cognitive deficits. Recent sub-analysis of phase 3 RCT data shows poorer high-order neurological functions in dexamethasone-treated patients with *recurrent* GBM (30). Dexamethasone also leads to significant mobility impairment in GBM patients (31).

Perioperative dexamethasone use leads to its dependence during adjuvant therapy, which lowers survival. Retrospective analyses of large cohorts and *post hoc* analysis of two large, multicenter, phase 3 RCTs show that GBM patients treated with dexamethasone during chemoradiation have lower survival (32–35). Thus, the use of dexamethasone *during* chemoradiation has declined (36). However, GBM patients are often prescribed huge amounts of dexamethasone (>160 mg over 3 weeks) perioperatively before adjuvant chemoradiation (11, 12). And, our data show greater perioperative dexamethasone use leads to greater use of dexamethasone during chemoradiation, even in patients with a gross total GBM resection (11).

Perioperative dexamethasone increases GBM malignancy and resistance before adjuvant therapy. Dexamethasone enhances the proliferative and migrative capacities of GBM (37, 38) and makes it resistant to chemoradiation (39–43). For example, in a murine GBM model, dexamethasone administered daily for 3 days before radiation significantly lowered the survival (33).

Perioperative dexamethasone weakens the systemic immunity of GBM patients before adjuvant therapy. dexamethasone is 30x more potent an immunosuppressant than endogenous steroids (13, 44). Routine preoperative treatment with dexamethasone (commonly 4 mg three times a day for 7 days) significantly depletes B cells, T cells, and NK cells in the blood of newly diagnosed GBM patients before surgery (45, 46). Up to 30% of GBM patients treated with dexamethasone have blood CD4 T cell counts < 200/mm (3) before surgery (i.e., they meet AIDS criteria)! (45, 47). Postoperatively, GBM patients receive even greater doses of dexamethasone (6-16 mg daily for 1-2 weeks)! which further worsens lymphopenia before starting chemoradiation (11, 12). Lymphopenia at the start or during chemoradiation predicts a lower survival in GBM patients (11, 32, 48–50). Not surprisingly, higher doses of perioperative dexamethasone lead to more infections in GBM patients in the 90 days after surgery (11, 51, 52). Infections cause treatment delays, are a direct cause of death (53, 54), and are an independent predictor of survival in GBM patients (11). Perioperative dexamethasone is a well-established cause of infections in other neurosurgery patients (55, 56). A large, multicenter, phase 3 RCT showed significantly greater infections in subdural hematoma neurosurgery patients randomized to a 2-week course of dexamethasone (29). A similar 2-week dexamethasone course is commonly prescribed postoperatively to GBM patients, who unlike other neurosurgery patients become further immunosuppressed by chemoradiation.

Perioperative dexamethasone may deplete GBM-infiltrated immune cells before adjuvant therapy (57). Among cancers, GBM is uniquely depleted in lymphocytes (58). Although it is accepted as GBM’s natural state due to the brain’s immune privilege (59), this idea is likely confounded by dexamethasone. GBM patients are the *only* cancer patients who receive dexamethasone before surgery, which causes lymphocyte apoptosis (60). Data of *recurrent* GBMs from dexamethasone-treated patients support this hypothesis (61). Higher GBM-infiltrated T cells directly correlate with longer survival in GBM patients (62).

Perioperative dexamethasone may attenuate immunotherapy. Cancer immunotherapies activate immunity against cancer cells resulting in durable objective responses (63, 64). Dexamethasone hinders them (65). In murine GBM models, it eradicates the survival benefit of immune checkpoint inhibitors and oncolytic viral therapy by depleting T cells and blocking potent intratumoral and global immune responses (66, 67). Retrospective results in GBM patients are similar (66). Moreover, recent large, multicenter, multinational, phase 3 RCTs of immune checkpoint inhibitors in GBM patients were not efficacious (2, 9, 10). However, subgroup analysis of one trial showed efficacy in those who did not receive dexamethasone! (10) In another phase 2 RCT that failed to show the efficacy of a checkpoint inhibitor, 90% of the efficacious responses occurred in GBM patients who did not receive dexamethasone (68). In a neo-antigen vaccine trial in GBM patients, dexamethasone prevented favorable neo-antigen-specific T cell responses and infiltration of T cells in the tumor (69).

Last, dexamethasone clouds the response assessment for high-grade gliomas (RANO-HGG). Dexamethasone affects GBM’s radiographic visibility with contrast and its use is a criterion in clinically determining GBM progression (36, 70, 71). How dexamethasone affects RANO-HGG is unclear. For example, it is unknown if perioperative dexamethasone use leads to an increase in progression-free survival (i.e., “pseudoresponse”), as seen with drugs that block the cerebral vasculature, like anti-VEGF drugs, to decrease tumor visibility on radiocontrast-based imaging (70). Dexamethasone use is also a criterion in the response assessment for high-grade gliomas (RANO-HGG) (36, 70, 71). On the other hand, we have shown that higher perioperative Dex doses lead to dexamethasone use during adjuvant therapy (11). Thus, it could also be likely that patients treated with high doses of dexamethasone may be judged to have shorter progression-free survival.

## Perioperative dexamethasone use in current practice

Perioperative dexamethasone use remains high despite rigorous evidence and emerging concerns for its harm (23). Our retrospective study of 360 GBM patients showed that greater perioperative dexamethasone independently correlates with lower survival (11). This result is independent of tumor size and has been replicated at other major cancer centers (72, 73). It is paradoxical to expect a survival benefit in trials of new adjuvant therapies, especially immunotherapy, while pre-treating patients with high doses of a potent therapy-nullifying steroidal immuno-suppressant. Despite emerging reports of its harm, a 2022 worldwide survey showed that 80% of neurosurgeons use dexamethasone perioperatively, and 45% use it liberally irrespective of patient symptoms (74). A group of multidisciplinary experts have recently questioned the perioperative use of high doses of dexamethasone (75). To best drive a practice change, data from a well-designed, rigorous study are needed.

## Alternatives to high-dose perioperative dexamethasone

To generate rigorous evidence that informs the practice of perioperative dexamethasone, prospective comparative studies are necessary. The comparator to the regular 2-week high-dose dexamethasone taper can be no dexamethasone use, limited use of dexamethasone [justified here (75)], or an alternative to dexamethasone [for example, RAGE inhibitors (76)]. Given the harms of dexamethasone, some providers may think there is equipoise in not giving dexamethasone perioperatively altogether, especially considering that dexamethasone is not a proven treatment to reduce seizures, intracranial pressure in a similar manner to mannitol or hypertonic saline, and disability caused by hemorrhage or post-operative stroke (in fact, there is data that argue it is harmful in stroke). In the perioperative setting, dexamethasone is only believed to *mitigate* (not eradicate) symptoms from the compressed and edematous peri-tumoral brain—not the tumor-harboring brain. GBM resection provides equal or greater symptom control by lowering brain pressures. Neurosurgeons have surgically treated GBM patients without perioperative dexamethasone (72, 74), and a trial of not using perioperative dexamethasone in GBM patients is currently underway to rigorously test its safety (NCT04266977).

## Conclusion

Dexamethasone has been used as part of the treatment for brain tumor patients since the 1960s including using it perioperatively (21). It is used with no rigorous evidence (22–24). While dexamethasone offers recognizable short-term benefits to the patients, the literature is increasingly pointing to it as a source of perioperative complications (52, 77) and long-term harms as well as an impediment to treatments and their response assessments. Collectively, the growing data mandate rigorous testing of the benefits of using perioperative dexamethasone.

## Data availability statement

The original contributions presented in the study are included in the article/supplementary material. Further inquiries can be directed to the corresponding author.

## Author contributions

AM: Writing – original draft, Writing – review & editing.
